# A System of Practical Medicine, Comprised in a Series of Original Dissertations

**Published:** 1841-07-17

**Authors:** 


### MEDICAL EXAMINER.

DEVOTED TO MEDICINE, SURGERY, AND THE COLLATERAL SCIENCES.

No. 29.] PHILADELPHIA, SATURDAY, JULY 17, 1841. [Vol. IV.

### BIBLIOGRAPHICAL NOTICE.

*A system of Practical Medicine, comprised in a series of original dissertations.* Arranged and edited by Alexander Tweedie, M. D., F. R. S., Fellow of the Royal College of Physicians, &c. Philadelphia: Lea & Blanchard, 1841.

This volume completes the series of works known under the name of Tweedie's Library of Practical Medicine. Its contents are necessarily of a miscellaneous character, and include those diseases which are not capable of systematic grouping under the title of any particular cavity of the body. These are hæmorrhage, dropsy, gout, rheumatism, scurvy, &c.; and lastly, a formulary of prescriptions. The treatises on these diseases are quite equal to those contained in the preceding volumes, but the formulary, although well enough in its way, is still far from equal to the other parts of the work. It is not without its immediate use to the young practitioner, although the ultimate benefit of such an aid may well be questioned.

The articles on hæmorrhage are by Dr. Burrows, and embrace a very full, but still concise description of this class of disorders, both as simple affections, or complications occurring during the course of various diseases. The proper stress is laid upon the secondary nature of many cases of hæmorrhage, which is totally neglected in most works upon this subject.

Dropsy is another of those groups in the present volume treated by Dr. Watson: these are secondary in most cases, and have, therefore, been already noticed in the articles relating to the organic diseases which give rise to them; still they require a distinct grouping or classification, for the symptoms sometimes become so prominent as to conceal, as it were, the original or primary disease.

Scurvy, by Dr. Budd, and scrofula, by Dr. Shapter, are both capital articles, especially the former. Rheumatism is a more difficult subject to treat, and the paper on the subject, by Dr. Budd, does not please us quite as well.

The Library being now complete, forms an excellent collection of works on the practice of medicine, intended for easy and convenient access, and bringing the subjects treated of down to the present day.

